# Clock and clock-controlled genes are differently expressed in the retina, lamina and in selected cells of the visual system of *Drosophila melanogaster*

**DOI:** 10.3389/fncel.2015.00353

**Published:** 2015-09-15

**Authors:** Milena Damulewicz, Agnieszka Loboda, Karolina Bukowska-Strakova, Alicja Jozkowicz, Jozef Dulak, Elzbieta Pyza

**Affiliations:** ^1^Department of Cell Biology and Imaging, Institute of Zoology, Faculty of Biology and Earth Sciences, Jagiellonian UniversityKrakow, Poland; ^2^Department of Medical Biotechnology, Faculty of Biochemistry, Biophysics and Biotechnology, Jagiellonian UniversityKrakow, Poland; ^3^Department of Clinical Immunology and Transplantology, Polish-American Institute of Pediatrics, Medical College, Jagiellonian UniversityKrakow, Poland

**Keywords:** circadian rhythms, photoreceptors, visual interneurons, glial cells, BRP synaptic protein, sodium pump, PDF receptors

## Abstract

The retina and the first optic neuropil (lamina) of *Drosophila* show circadian rhythms in various processes. To learn about the regulation of circadian rhythms in the retina and lamina and in two cell types, glial and the lamina L2 interneurons, we examined expression of the following clock genes; *per*, *tim*, *clk*, and *cry* and clock-controlled genes (ccgs); *Atp*α, *nrv*2, *brp, Pdfr*. We found that the expression of gene studied is specific for the retina and lamina. The rhythms of *per* and *tim* expression in the retina and glial cells are similar to that observed in the whole head and in clock neurons, while they differ in the lamina and L2 cells. In both the retina and lamina, CRY seems to be a repressor of *clk* expression. In L2 interneurons *per* expression is not cyclic indicating the other function of PER in those cells than in the circadian molecular clock. In contrast to *per* and *tim*, the pattern of *clk* and *cry* expression is similar in both the retina and lamina. The retina holds the autonomous oscillators but the expression of *cry* and ccgs, *Atp*α and *nrv*2, is also regulated by inputs from the pacemaker transmitted by PDF and ITP neuropeptides.

## Introduction

The circadian clock is a self-sustaining generator of endogenous oscillations with a period of about a day that generates rhythmicity in biochemical and physiological processes of most organisms and in the behavior of animals. It maintains the homeostasis of organisms in time, anticipates daily changes of light in the environment and its function continues in constant darkness (DD). Under day/night or light/dark (LD) conditions the circadian clock is synchronized by light and the period of the rhythm is equal to 24 h. In DD the period becomes longer or shorter than 24 h.

In animals, circadian rhythms are mostly studied in terms of behavior, predominantly in locomotor activity, but they can also be observed in the activity and structure of cells, including neurons and glial cells (Pyza, 2010). In the visual system of flies, the morphology of cells, synaptic proteins and activity of ion pumps oscillate during the day and most of these rhythms are correlated with the species specific rhythm in locomotor activity. These cyclical changes also occur in DD, indicating that remodeling of the neuron structure and synaptic contacts in the day is regulated by the circadian clock. The changes in morphology of neurons and synapses are synchronized with animal activity and with daily changes of light intensity in the environment (Weber et al., 2009; Damulewicz et al., 2013; Górska-Andrzejak et al., 2005, 2013).

The visual system of the fruit fly *Drosophila melanogaster* provides an excellent model for studying circadian rhythms at the cellular level. It consists of the retina and three optic neuropils: lamina, medulla and lobula. The retina is composed of 700–800 single modules called ommatidia and each ommatidium comprises eight photoreceptor cells, R1–R8. Six of them, R1–R6 terminate in the first optic neuropil (lamina) while R7 and R8 pass the lamina and terminate in the medulla (Meinertzhagen and Hanson, 1993). The photic and visual information received by the retina photoreceptors are transmitted to the lamina by tetrad synapses formed between R1–R6 and the first order lamina interneurons, large monopolar cells L1 and L2 and two other cell types (Meinertzhagen and O’Neil, 1991). Like the retina, the lamina also has a modular structure and is composed of cylindrical units called cartridges. Each cartridge is surrounded by three glial cells and is composed of six photoreceptor terminals, five lamina monopolar cells, processes of amacrine cells and axons of neurons located in other visual neuropils and in the central brain (Meinertzhagen and Sorra, 2001). The lamina not only receive an efferent input from the retina through tetrad synapses but also sends feedback synapses back to the photoreceptor terminals R1–R6.

Circadian rhythms have been detected in both the retina and lamina of flies. In the retina, circadian oscillations have been found in the amplitude of the electroretinogram (ERG) and synthesis of photopigment (Chen et al., 1992). In the lamina, the number of tetrad and feedback synapses (Pyza and Meinertzhagen, 1993) and the size of monopolar cells L1 and L2 and glial cells (Pyza and Meinertzhagen, 1995, 1999; Pyza and Górska-Andrzejak, 2004) oscillate during the day and night.

The rhythms in the lamina are generated by circadian oscillators located in the brain, by the so-called central clock or pacemaker and by peripheral oscillators located in the retina photoreceptors and in some glial cells of the optic lobe (Damulewicz et al., 2013; Górska-Andrzejak et al., 2013). The central clock consists of about 150 clock cells, expressing clock genes, located in the proximal medulla, namely: ventral lateral neurons (LN_v_s) and dorsal lateral neurons (LN_d_s) and in the dorsal protocerebrum, three groups of dorsal neurons (DN_s1–3_). The LN_v_s, except the so-called 5th small LN_v_, express the neuropeptide pigment-dispersing factor (PDF), while the 5th small LN_v_ and one of the LN_d_s express the ion transport peptide (ITP; Johard et al., 2009). The 5th small LN_v_ projects to the lamina and this projection is immunoreactive to ITP, however, both peptides, PDF and ITP, are involved in regulating circadian rhythms in the lamina (Damulewicz and Pyza, 2011).

The circadian rhythms in the retina seem to be generated by circadian oscillators located in photoreceptors, as they expressed the clock genes *period* (*per*), *timeless* (*tim*; Siwicki et al., 1988; Zerr et al., 1990) and *cryptochrome* (*cry*; Yoshii et al., 2008). It has been suggested that the retina possesses an autonomous clock, independent from the pacemaker located in the brain (Siwicki et al., 1988; Cheng and Hardin, 1998). Moreover, in the retina photoreceptors *per* mRNA peaks later during the day than in the pacemaker and PER protein is degraded earlier in the day (Zerr et al., 1990). Nothing is known, however, about expression of clock genes in the lamina, a site of pronounced circadian rhythms in changes of synapse frequency, neurons and glial cell morphology and of protein level. It is also unknown how molecular clocks operate in the retina and in glial cells that express *per* and other clock genes.

To learn how the rhythms in the retina and lamina are regulated we examined the expression of clock genes and possible clock-controlled genes (ccgs) in the retina or lamina, isolated from the *Drosophila* head manually or by laser microdissection. We studied the expression of two core genes of the molecular clock, *per* and *tim*, as well as *clock* (*clk*), encoding the transcription factor CLK, that together with CYCLE (CYC), activates the transcription of *per*, *tim* and ccgs. We also examined the expression of the *cry* gene, a circadian photoreceptor in most of the clock neurons and a circadian transcriptional repressor of the molecular clock in the peripheral oscillators (Stanewsky et al., 1998; Collins et al., 2006). In addition to clock genes we examined the expression of the following ccgs; *Atp*α, *nrv2* and *brp*. *Atp*α and *nrv2* encode α and β subunits of the Na^+^/K^+^-ATPase, a major ion pump in all cells and *brp* encodes the presynaptic scaffolding protein Bruchpilot (BRP). ATPα and BRP show strong circadian rhythms in abundance in the lamina (Damulewicz et al., 2013; Górska-Andrzejak et al., 2013). The expression of these genes was examined at four time points in wild-type flies maintained in light/dark (LD12:12) or constant darkness (DD) conditions, in null mutants of *per* (except the *per*), *cry* (except the *cry*), *Pdf* and in a strain with decreased expression of *itp* in LD 12:12.

In addition, the expression of *per*, *tim*, *Atpα*, *brp* and *Pdfr—*encoding PDF receptors, was studied at four time points in LD 12:12 in two types of cells; the lamina L2 monopolar cells, the first order interneurons in the visual system, and glial cells, which show robust circadian rhythms in structural changes. In addition to checking the rhythms in the mRNAs of clock genes and ccgs in two different layers of the visual system and in two cell types, we also tested if those rhythms are affected by the lack of CRY or clock neurotransmitters PDF or ITP.

The expression of clock and ccgs have not been studied in the visual system—a site of circadian plasticity and in glial cells that participate in the regulation of circadian rhythms in the brain and in behavior (Jackson, 2011; Górska-Andrzejak, 2013).

## Materials and Methods

### Animals

The following strains of *D. melanogaster* were used: wild-type Canton S, *cry*^01^—a null mutant of the circadian photoreceptor gene *cryptochrome* (Dolezelova et al., 2007), *Pdf* ^0^—a null mutant of the clock neuropeptide *Pigment dispersing factor* gene, *per*^01^—null mutant of the clock gene *period*, *cry*-*GAL4*.*39* expressing the yeast transcription factor GAL4 under control of *cry* promoter, *21D-GAL4/MKRS* expressing GAL4 in L2 interneurons, *repo-GAL4* expressing GAL4 under control of *repo* (*reversed polarity* gene) promoter, a specific marker of glial cells, UAS-*dicer2*; UAS-*itp-RNAi/*MKRS, expressing interfering RNA for *itp* (ITP gene) and *dicer2* which catalyses the first step of RNA interference, under control of the UAS sequence, and *UAS-gfp* expressing Green Fluorescent Protein (GFP) under control of the UAS sequence. The following crosses were carried out: *cry-GAL4* × UAS-*dicer2*; UAS-*itp-RNAi/*MKRS; *21D-GAL4* × UAS*-gfp* and *repo-GAL4* × UAS*-gfp*. The first generation of *cry > itpRNAi* strain has the *itp* expression silenced only in two cells: in one of the LN_d_s and in the 5th small ventral lateral neuron (s-LN_v_). *21D* > *gfp* and *repo* > *gfp* strains were used to visualize L2 and glial cells, respectively, with GFP.

Flies were maintained on a standard cornmeal medium under a light/dark condition of LD12:12 (12 h of light and 12 h of darkness) or constant DD and at constant temperature 24°C.

### Laser Microdissection

Males, 7-days old, were decapitated at the following five time points: ZT1, ZT4, ZT13, ZT16, ZT20 (where ZT0 means light-on, ZT12 means light-off). In constant darkness flies were collected at CT1, CT4, CT13, CT16, CT20 under dim red light illumination. Approximately 30 flies were used for each time point and experiments were repeated at least three times. Heads were fixed in 96% ethanol for 45 min, then they were washed in phosphate buffer saline (PBS, pH 7.4) twice and cryoprotected by incubation in 12.5% sucrose for 10 min and in 25% sucrose at 4°C overnight. Next they were embedded in Tissue-Tek, frozen in liquid nitrogen, and cryostat 16 μm sections were cut and collected on special RNase, DNase—free membrane slides (PEN).

Laminas were selected, marked and dissected using an UV laser microdissector Leica LMD7000 (power: 15; aperture: 2; speed: 6; specimen balance 20; pulse frequency: 5000; S1 Figure). Specimens were collected using gravity into caps of microcentrifuge tubes containing a lysis buffer (NucleoSpin RNA XS kit, Macherey Nagel). Thirty laminas were collected for each time point. The retinas were cut manually from the optic lobe.

### Cell Sorting

Males of the *repo > gfp* and *21D > gfp* strains were decapitated at four time points: ZT1, ZT4, ZT13, and ZT16. Brains were isolated on ice and digested in trypsin-EDTA (Sigma) for 1 h in 37°C. Cells were centrifuged for 10 min at 10,000 rpm, after which the cell pellet was resuspended in PBS and incubated with Hoechst dye for 15 min. Then the cells were centrifuged for 10 min at 10,000 rpm and resuspended in PBS. A MoFlo XDP cytofluorimeter with a sorter (Beckman Coulter) was used for cell sorting (laser: 488 nm, 633 and 375 nm). Cells were sorted depending on size and granulation, Hoechst fluorescence and GFP signal. At least 1000 cells were isolated from one sample. 10 flies were used for each time point and experiments were repeated three times.

### RNA Isolation and Real Time PCR

Total RNA isolation was performed using a NucleoSpin RNA XS kit (Macherey Nagel) according to the manufacturer’s protocol. 500 ng of total RNA was used for reverse transcription and then 1:8 diluted cDNA was used for quantitative PCR. Gene expression was examined using TaqMan Gene Expression Assays with 6′-FAM as a labeling molecule (Applied Biosystems) and 7500 Fast Real-Time PCR System (Applied Biosystems). The following genes were examined: *period* (*per*, FBgn0003068, Assay ID: Dm01843683_g1), *timeless* (*tim*, FBgn0014396; Assay ID: Dm01814244_g1), *clock* (*clk*, FBgn0023076; Assay ID: Dm01795381_g1), *cryptochrome* (*cry*, FBgn0025680; Assay ID: Dm02149911_m1) *nervana2* (*nrv2*, FBgn0015777; Assay ID: Dm01803292_g1), *sodium pump α-subunit* (*Atpα*, FBgn0002921; Assay ID: Dm02154298_m1), *bruchpilot* (*brp*, FBgn0259246; and Assay ID: Dm01794336_g1). Expression of the *Pdfr* (FBgn0260753) gene was examined using SybrGreen qPCR (Applied Biosystem; forward primer: 5′ACGGCCGTGTTACAAGCCG, reverse primer: 5′GGAGAGGCAGAGGCCCACGA). Each experiment was repeated at least three times.

Data were collected as raw C_T_ values and analyzed using the 2^−ΔΔ CT^ method. *Ribosomal protein 32* (*rpl32*, FBgn0002626; Assay ID: Dm02151827_g1 or forward primer: 5′AGAAGCGCAAGGAGATTGTC and reverse primer: 5′ATGGTGCTGCTATCCCAATC) was used as a reference gene. Gene expression was normalized on an arbitrary scale with ZT1 as 1.0.

Statistical analysis was performed using initially the non-parametric ANOVA Kruskal-Wallis test and then Tukey’s test. Statistica 7.0 software was used for the analyses. GraphPad Prism was used to prepare Figures.

## Results

### Gene Expression in the Retina

**Figure 1:**
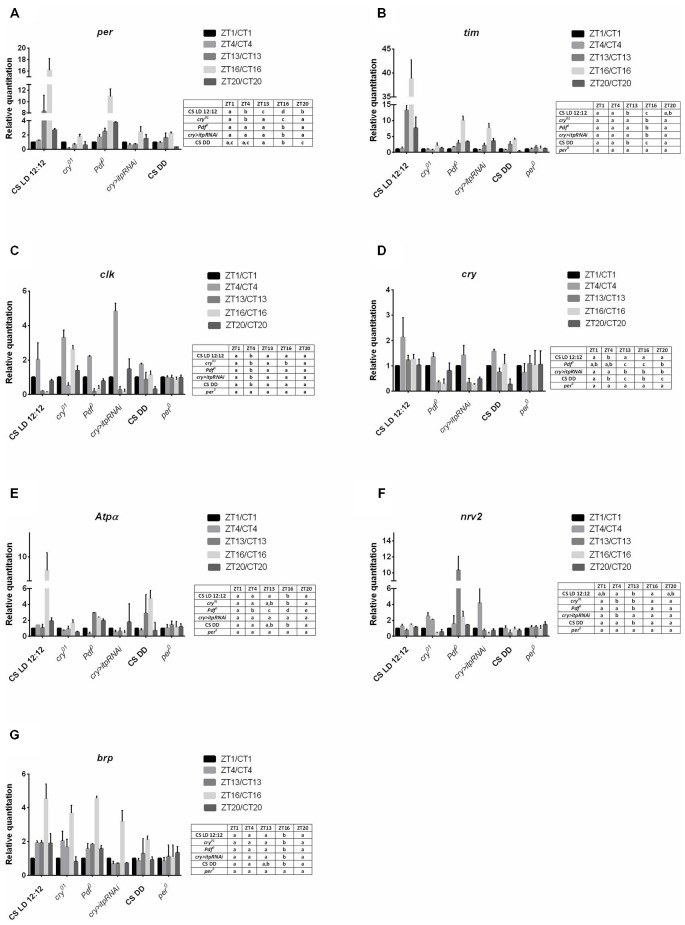
**Gene expression in the retina of different strains of *D. melanogaster***. **A**—*per* gene; **B**—*tim*; **C**—*clk*, **D**—*cry*, **E**—*Atpα*, **F**—*nrv2*, **G**—*brp*. Tissues were collected at five time points: ZT1, ZT4, ZT13, ZT16, ZT20 in LD12:12 and CT1, CT4, CT13, CT16, CT20 in DD. Relative quantitation (RQ) determines the changes in steady-state mRNA level, data are normalized to ZT1 in LD 12:12 or CT1 in DD (value = 1.0). Gene expression was examined in LD 12:12 light conditions for Canton S, *cry*^01^, *Pdf* ^0^, *cry*>*itp-RNAi*, *per*^01^ and in constant darkness (DD) for Canton S. Statistical analyses were carried out to compare differences between time points for each genotype in the same condition. Statistically significant differences are indicated with small letters **(A–D)** in tables next to the figures. The same letter means no significant differences between those time points.

In the retina of wild-type flies maintained in LD 12:12, the expression of both core clock genes, *per* and *tim*, was cyclical with a peak reached at ZT16 but higher level of both gene mRNAs than in other time points was also detected at ZT13 (Figures 1A,B). In the *cry*^01^, *Pdf* ^0^ mutants and *cry > itpRNAi* strain, the daily expression of *per* and *tim* was maintained with the peak at ZT16, however, in contrast to Canton S the increase of mRNA levels at ZT13 was not observed. In all strain studied, except Canton S in LD 12:12 and *Pdf* ^0^, the amplitude of the *per* and *tim* mRNA rhythms was small (Table 1). The most striking changes in *per* and *tim* mRNA cycling were observed in the *cry*^01^ mutant and *cry > itpRNAi* strain, indicating that CRY and ITP are important for maintaining high levels of *per* and *tim* mRNAs during the night. In *per*^01^ mutant the expression of *tim* was arrhythmic (Figure 1B). In DD the pattern of *per* and *tim* gene expression in the retina of Canton S flies was similar to their expression in LD12:12 with a peak at CT16 but the amplitude of the rhythms was low (Figures 1A,B).

**Table 1:** **Amplitudes of the daily rhythms of clock and clock-controlled gene mRNAs in the retina**.

Gene	Strain	Amplitude
*per*	Canton S LD 12:12	16.13 ± 2.89
	*cry^01^*	1.52 ± 0.63
	*Pdf^ 0^*	10.07 ± 2.05
	*cry > itpRNAi*	1.86 ± 1.39
	Canton S DD	1.89 ± 0.21
*tim*	Canton S LD 12:12	38.31 ± 0.78
	*cry^01^*	1.17 ± 0.27
	*Pdf^ 0^*	9.32 ± 0.43
	*cry > itpRNAi*	6.73 ± 1.24
	Canton S DD	3.61 ± 0.24
*clk*	Canton S LD 12:12	1.88 ± 0.28
	*cry^01^*	2.68 ± 0.61
	*Pdf^ 0^*	2.17 ± 0.32
	*cry > itpRNAi*	4.67 ± 1.55
	Canton S DD	2.11 ± 0.46
*cry*	Canton S LD 12:12	1.12 ± 1.09
	*Pdf^ 0^*	1.01 ± 0.53
	*cry > itpRNAi*	1.22 ± 0.31
	Canton S LD 12:12	1.3 ± 0.23
*Atpα*	Canton S LD 12:12	8.43 ± 1.04
	*cry^01^*	1.11 ± 0.71
	*Pdf^ 0^*	2.65 ± 0.26
	*cry > itpRNAi*	0.58 ± 0.39
	Canton S DD	4.0 ± 1.28
*nrv2*	Canton S LD 12:12	0.71 ± 0.44
	*cry^01^*	2.11 ± 0.79
	*Pdf^ 0^*	9.29 ± 2.53
	*cry > itpRNAi*	3.78 ± 0.82
	Canton S DD	0.8 ± 0.1
*brp*	Canton S LD 12:12	3.52 ± 0.89
	*cry^01^*	2.88 ± 0.62
	*Pdf^ 0^*	3.56 ± 0.13
	*cry > itpRNAi*	2.51 ± 0.79
	Canton S DD	1.29 ± 0.29

The expression of *clk* in the retina was also cyclic and the pattern was the same in Canton S, (in both LD 12:12 and DD conditions), *Pdf* ^0^ and *cry > itpRNAi*, reaching its peak at ZT4 (Figure 1C). However, this pattern was changed in the *cry*^01^ mutant. In addition to a peak of *clk* mRNA at ZT4, the second one was observed at ZT16. It suggests that CRY suppresses *clk* expression at ZT16. In *per*^01^ mutant the level of *clk* mRNA was the same at four time point studied.

In the retina, *cry* is regarded as a circadian photoreceptor and an element of the molecular circadian clock (Collins et al., 2006). In wild-type flies the *cry* expression was the highest at ZT4 and a similar level was observed at other time points (Figure 1D). In *Pdf* ^0^ and *cry > itpRNAi* strains, this peak was also observed, however, in *Pdf* ^0^ the second peak was at ZT20. The amplitude of the *cry* mRNA rhythm was similar in all the strains studied (Table 1). In *per*^01^ mutant *cry* expression was arrhythmic. In DD the rhythm was maintained with peaks at CT4 and CT16. The obtained results suggest that light and PDF, which is released from terminals of l-LN_v_s, affect *cry* expression in the retina.

The daily expression of two genes, *Atpα* and *nrv2*, encoding the α and β subunits of Na^+^/K^+^-ATPase, was detected in the retina but its pattern was specific for each gene (Figures 1E,F). The α subunit gene (*Atpα*) expression peaked at ZT16 while that of *nrv2* showed two peaks at ZT4 and ZT16. However, at ZT1 and ZT20 the level of *nrv2* mRNA was only slightly lower than at ZT4 and ZT16. In case of *Atpα* the pattern observed in Canton S was unchanged in the *cry*^01^ mutant (Figure 1E), but the amplitude of this rhythm was very low (Table 1). In contrast, the daily pattern of *nrv2* mRNA was completely different in the *cry*^01^ mutant because the highest level of mRNA was at ZT4 and was still high at ZT13, when compared with other time points (Figure 1F). When both gene mRNAs were studied in the *Pdf* ^0^ and *cry > itpRNAi* strains their daily patterns were deemed to have changed. In the *Pdf* ^0^ mutant, the peak of *Atpα*mRNA was found at ZT13 and a high level of mRNA was also maintained throughout the rest of the night (Figure 1E). The amplitude of the *Atpα*mRNA rhythm in the *cry*^01^ and *Pdf* ^0^ flies was small (Table 1). In the *cry > itpRNAi* strain the rhythm of *Atpα* mRNA was abolished (Figure 1E). The mRNA of the *nrv2* gene showed daily oscillations with a peak at ZT13 in the *Pdf* ^0^ mutants (Figure 1F). The pattern of this rhythm was different than that found in Canton S, and the *cry*^01^ and *cry > itpRNAi* strains. In *cry > itpRNAi* flies, the daily rhythm of *nrv2* was also observed although the peak was determined at ZT4 and this pattern was different than that present in other strains (Figure 1F). In *cry*^01^, *Pdf* ^0^ and *cry > itpRNAi*, the amplitude of the *nrv2* mRNA rhythm was larger than in Canton S (Table 1). In *per*^01^ mutant the expression of both *Atpα* and *nrv2* genes was arrhythmic. In DD conditions *nrv2* mRNA showed a trough at CT13 and *Atpα* had the similar pattern of expression as in LD 12:12 with a peak at CT16. The obtained results indicate that in the retina the expression of *Atpα* and *nrv2* is cyclic and controlled by clock genes. Moreover CRY, PDF and ITP affect both rhythms.

The expression of *brp* gene in the retina of wild type flies was cyclic with the maximum found in the middle of the night at ZT16 and at CT16 in DD (Figure 1G). This pattern did not change in other experimental strains, except of *per*^01^ mutant. In this mutant the circadian rhythm of *brp* expression was abolished. The obtained results suggest that neither CRY nor neuropeptides PDF and ITP affect the circadian rhythm of *brp* expression in the retina.

### Gene Expression in the Lamina

**Figure 2:**
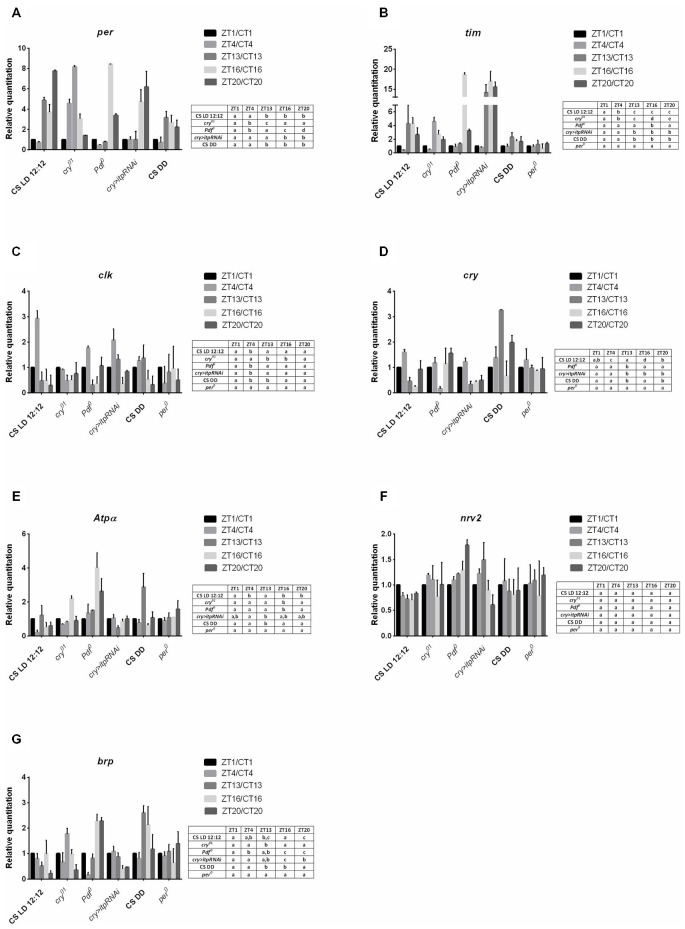
**Gene expression in the lamina of different strains of *D. melanogaster*. A**—*per* gene; **B**—*tim*; **C**—*clk*, **D**—*cry*, **E**—*Atpα*, **F**—*nrv2*, **G**—*brp*. Data are normalized to ZT1 or CT1 (value = 1.0). The following strains were examined in LD 12:12: Canton S, *cry*^01^, Pdf ^0^, cry > itp-RNAi, *per*^01^ and in DD: Canton S. Statistically significant differences are shown in tables next to the figures.

In wild type (Canton S) flies all the studied gene expression was detected in the lamina. All of them, except *nrv2*, also showed rhythmic mRNA changes in both LD 12:12 and DD. The expression of *per* and *tim* in the lamina was significantly higher during the night than during the day (Figures 2A,B) but the amplitude of *per* mRNA oscillations was high in comparing with that of *tim* (Table 2). In the *cry*^01^ mutant *per* and *tim* mRNA levels were the highest at ZT13 but the amplitude of the rhythm was high for *per* but low for *tim*. Moreover, in contrast to Canton S, the mRNA level of *per* was additionally high at ZT4 and low at ZT20 (Figure 2A). In the *Pdf* ^0^ mutant, which does not synthesize the clock neuropeptide PDF we observed a different pattern of *per* and *tim* expressions compared with wild-type flies. This strain showed maxima of *per* and *tim* mRNAs at ZT16 that were several times higher than at other time points (Figures 2A,B). After silencing *itp* using the *cry-GAL4* > UAS*-itpRNAi* strain, the high level of *per* mRNA was found at ZT16 and ZT20 and of *tim* mRNA at ZT13, ZT16 and ZT20. The lack of PDF or reduced level of ITP strongly increased *per* and *tim* expressions during the night at ZT16. In both the *Pdf* ^0^ mutant and *cry-GAL4* > UAS*-itpRNAi* strain, the daily pattern of *per* and *tim* expressions was similar in the lamina and retina. The expression of *tim* was arrhythmic in *per*^01^ mutant (Figure 2B). In DD conditions the expression of *per* and *tim* genes was similar as in LD 12:12 (Figures 2A,B) but the amplitude of oscillations was decreased (Table 2) in comparing with other strain studied.

**Table 2:** **Amplitudes of the daily rhythms of clock and clock-controlled gene mRNAs in the lamina**.

Gene	Strain	Amplitude
*Per*	Canton S LD 12:12	7.04 ± 2.98
	*cry^01^*	6.43 ± 0.32
	*Pdf^ 0^*	7.98 ± 0.1
	*cry > itpRNAi*	5.95 ± 2.35
	Canton S DD	2.64 ± 0.62
*Tim*	Canton S LD 12:12	3.83 ± 0.57
	*cry^01^*	4.35 ± 1.16
	*Pdf^ 0^*	9.21 ± 0.31
	*cry > itpRNAi*	18.35 ± 4.42
	Canton S DD	1.25 ± 0.94
*Clk*	Canton S LD 12:12	3.13 ± 1.65
	*cry^01^*	0.52 ± 0.15
	*Pdf^ 0^*	1.64 ± 0.13
	*cry > itpRNAi*	1.79 ± 0.26
	Canton S DD	1.69 ± 0.22
*Cry*	Canton S LD 12:12	1.42 ± 0.19
	*Pdf^ 0^*	1.01 ± 0.6
	*cry > itpRNAi*	0.9 ± 0.36
	Canton S DD	2.24 ± 1.1
*Atpα*	Canton S LD 12:12	0.79 ± 0.14
	*cry^01^*	1.61 ± 0.11
	*Pdf^ 0^*	3.01 ± 0.98
	*cry > itpRNAi*	0.58 ± 0.48
	Canton S DD	2.24 ± 0.75
*Brp*	Canton S LD 12:12	0.74 ± 0.1
	*cry^01^*	1.42 ± 0.59
	*Pdf^ 0^*	2.12 ± 0.35
	*cry > itpRNAi*	0.69 ± 0.47
	Canton S DD	1.97 ± 0.67

The *clk* expression was cyclic in the lamina of the wild-type, *cry*^01^, *Pdf* ^0^ and *cry > itpRNAi* flies. Moreover, the daily pattern of changes was similar in all strains studied, except the *cry*^01^ mutant. In Canton S, *Pdf* ^0^ and *cry > itpRNAi*, the mRNA level of this gene was the highest at ZT4 (Figure 2C) although the amplitude of the rhythms was lower in *Pdf* ^0^ and *cry > itpRNAi* than in Canton S (Table 2). In the *cry*^01^ mutant the *clk* expression pattern was completely changed. The higher level of mRNA was observed at ZT1, ZT4 and ZT20. The amplitude of this rhythm was low, since the mRNA level was only two times lower at ZT13 and ZT16 than at ZT1 (Table 2). The daily rhythm of *clk* expression was similar in the lamina and retina in all strains studied, except *cry*^01^. In *per*^01^ mutant the rhythm in changes of *clk* mRNA level was not detected. In DD mRNA level of *clk* was the highest at CT4 and CT13 and the amplitude of the circadian rhythm was low.

The mRNA level of *cry* also oscillates in the lamina in both LD 12:12 and DD. In wild-type flies the mRNA level of *cry* was the highest at ZT4, about nine times higher than in a trough at ZT16 (Figure 2D). In DD the expression pattern was changed with two peaks at CT13 and CT20 and similar level at CT1, CT4 and CT16. The amplitude of the rhythm was higher in DD than in LD 12:12. In the *Pdf* ^0^ mutant this pattern was different than in Canton S in LD 12:12 since the mRNA level was similar at the four time points studied but dropped significantly at ZT13. In turn in *cry > itpRNAi* flies the *cry* expression pattern was similar to that observed in wild-type flies with higher level of mRNA noted at ZT1 and ZT4. The daily pattern of *cry* expression was similar in the lamina and retina in Canton S and *cry > itpRNAi* flies but different in the *Pdf* ^0^ mutant. In *per*^01^ mutant the expression of *cry* was arrhythmic.

In addition to clock genes we also determined the expression of *Atpα*, *nrv2* and *brp* genes in the lamina, since they are involved in rhythmic processes in this optic neuropil. Examination of the mRNA level of both *Atpα* and *nrv2* genes in the lamina showed that *Atpα* mRNA oscillates during the day in Canton S, *cry*^01^, *Pdf* ^0^ and *cry > itpRNAi* flies (Figure 2E), but that the mRNA of *nrv2* is constant (Figure 2F). In case of *Atpα* the expression displayed the daily rhythm with two, morning (ZT1) and evening (ZT13) peaks (Figure 2E) but in DD the peak at CT1 was not detected and the amplitude of the rhythm was higher than in LD 12:12 (Table 2). In *cry*^01^ and *Pdf* ^0^ mutants only one peak was observed at ZT16, while after silencing of *itp* in the clock neurons, the level of *Atpα* mRNA was the same during the day and night, except ZT13 where it significantly dropped when compared with ZT4. The amplitude of the rhythm was the highest in *Pdf* ^0^. In *per*^01^ mutant *Atpα* expression was arrhythmic.

The expression of the *brp* gene was rhythmic in the lamina. In wild-type flies *brp* mRNA was high at ZT1, ZT4 and ZT16 and in DD at CT13 and CT16 (Figure 2G). In *cry*^01^ the peak of *brp* mRNA was located at ZT13 and the amplitude of the rhythm was larger than in wild-type flies (Table 2). In the *Pdf* ^0^ mutant and after silencing *itp*, the pattern of *brp* expression was different than in Canton S. The highest level of *brp* mRNA in *Pdf* ^0^ was observed at the end of the night, at ZT16 and ZT20 while in the *cry > itpRNAi* strain the high level was during the day at ZT4. In *per*^01^ mutant *brp* mRNA level was similar at four time point studied.

### Gene Expression in L2 Interneurons

**Figure 3:**
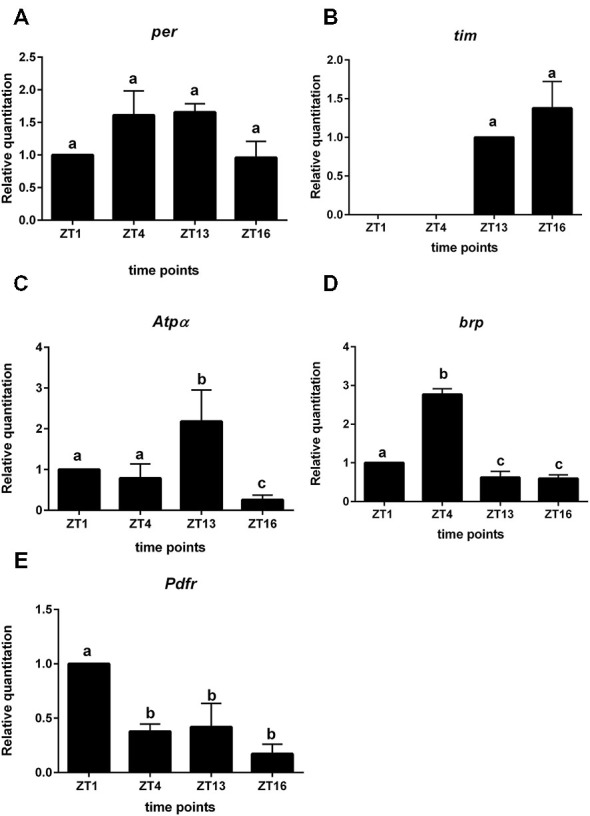
**Gene expression in L2 interneurons labeled with GFP (*21D > gfp* strain). A**—*per* gene; **B**—*tim*; **C**—*Atpα*, **D**—*brp*, **E**—*Pdfr*. Cells were isolated at four time points: ZT1, ZT4, ZT13, ZT16 in LD 12:12. RQ determines the changes in mRNA level and data are normalized to ZT1 (value = 1.0). Statistically significant differences are marked by different letters **(A–C)** above bars.

In sorted cells labeled with GFP in *21D > gfp* strain, the clock gene *per* was expressed but its mRNA level was constant during the day and night (Figure 3A). In turn *tim* expression was below the detection level during the day (ZT1, ZT4) but was higher during the night at ZT13 and ZT16 (Figure 3B). These results indicate the other functions of both clock genes in the lamina L2 monopolar cells rather than in photoreceptors and clock neurons. In turn the mRNA of the *cry* gene was not detected in L2 interneurons.

All non-clock genes, *Atpα*, *brp* and *Pdfr—*encoding PDF receptors, had cyclic expressions, although their mRNA daily patterns were specific for each gene (Figures 3C–E). The mRNA level of *Atpα* was the highest at ZT13 and the lowest at ZT16 (Figure 3C). The *brp* expression was rhythmic with the maximum detected at ZT4 (Figure 3D). The highest expression of *Pdfr* was observed at ZT1 and was more than two times higher than at other time points (Figure 3E). The mRNA of *nrv2* was not detected in L2 cells.

### Gene Expression in Glial Cells

**Figure 4:**
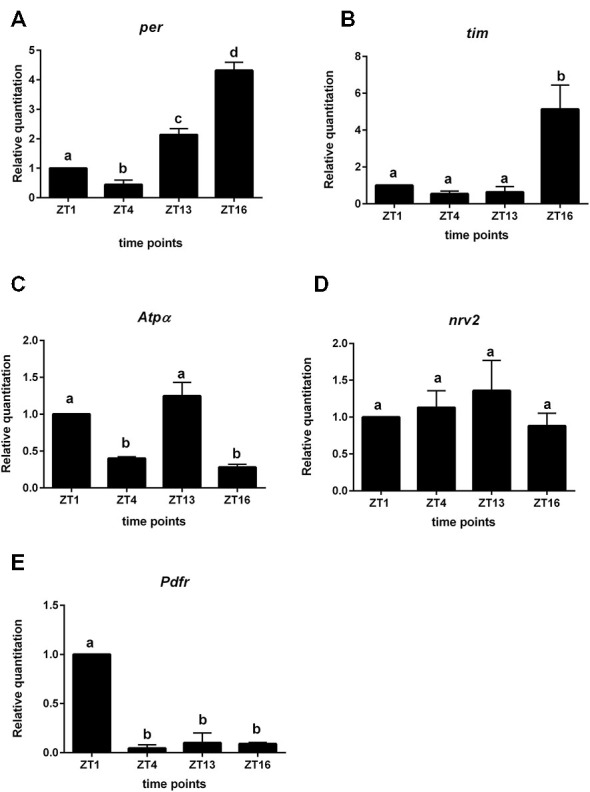
**Gene expression in glial cells labeled with GFP (*repo > gfp* strain). A**—*per* gene; **B**—*tim*; **C**—*Atpα*, **D**—*nrv2*, **E**—*Pdfr*. Cells were isolated at four time points: ZT1, ZT4, ZT13, ZT16 in LD 12:12. Data are normalized to ZT1 (value = 1.0). Statistically significant differences are marked by different letters **(A–C)** above bars.

We used the *repo > gfp* strain to obtain a purified population of glial cells from the brain of *Drosophila* by cell sorting. In these glial cells we detected oscillations of *per* and *tim* expression. The highest level of *per* mRNA was detected at ZT16 and the lowest at ZT4 (Figure 4A). This pattern was similar to that detected in the retina. The expression of *tim* peaked at ZT16 and was low during the day (ZT1, ZT4) and at the beginning of the night (ZT13; Figure 4B). The mRNA level of *cry* was very low, being almost at the limit of detection.

The expression of *Atpα* was rhythmic in glial cells, with two peaks, at ZT1 and ZT13. This pattern was similar to that obtained for the lamina (Figure 4C). The *nrv2* gene was expressed in glial cells but its expression was arrhythmic (Figure 4D). The mRNA of the *Pdfr* gene displayed the robust daily rhythm. The maximum of *Pdfr* mRNA was observed at ZT1 (Figure 4E), at the same time as in the L2 cells.

## Discussion

Studying the expression of clock genes and potential ccgs, in two layers of nerve cells in the visual system and in two types of cells which show circadian rhythms in activity and structural plasticity (Chen et al., 1992; Pyza, 2010), we found that during the day the clock core genes, *per* and *tim*, and also *clk* and *cry* were cyclically expressed in all tissue and cells studied except for *per* in the L2 interneurons. In these cells the expression of *per* was arrhythmic, suggesting that it plays another non-clock function. Moreover, in the L2 cells, *cry* expression was not detected and *tim* expression was different to that of *per* and it was below the detection level during the day but present during the night. In our earlier studies, we found that the L2 dendritic tree morphology does not oscillate and its shape is different in the *per*^01^ mutant (Weber et al., 2009), suggesting that PER in these cells may regulate the morphology of dendrites but their circadian plasticity is probably controlled by the retina clock, the pacemaker and glial oscillators as for other rhythms in the lamina (Damulewicz et al., 2013; Górska-Andrzejak et al., 2013).

The cyclic expression of gene studied was also maintained in constant darkness and abolished in *per^01^* mutant and this indicates that the expression of clock genes *per*, *tim*, *clk*, *cry* and ccgs *atpα, brp* is circadian in both the retina and lamina.

So far the expression of clock genes and other cycling transcripts have been studied only in whole head homogenates (Claridge-Chang et al., 2001; McDonald and Rosbash, 2001; Ceriani et al., 2002; Lin et al., 2002; Ueda et al., 2002) and in clock neurons, including LNs and DNs in the brain (Kula-Eversole et al., 2010; Nagoshi et al., 2010). However, the expression of clock genes in pacemaker cells or in the whole head does not explain how circadian rhythms in cellular processes and in structural plasticity, are regulated in different regions of the brain and in single neurons and glial cells. Our study is the first attempt to understand how circadian rhythms are regulated at the level of tissues and cells in the brain. Moreover, we successfully applied the laser microdissection method for the isolation of the lamina from the optic lobe of *D. melanogaster*, and also highlighted the fact that this method might be very valuable for other approaches as well.

In the fruit fly head homogenates, two peaks around ZT10 and ZT20 in the expression of *per*, *tim* and *clk*, *cry* have been detected (Ueda et al., 2002). In clock neurons the peak of *per* and *tim* mRNAs is at ZT16 (Kula-Eversole et al., 2010). In the present study, we found that the pattern of *per* and *tim* expression is similar in the retina and glial cells to that in the LN_v_s and maxima of *per* and *tim* expression are at ZT16 in LD 12:12 and CT16 in DD. In turn the expression of *clk* and *cry* peaks at ZT4/CT4. The rhythms of *per* and *tim* mRNAs in the retina were not affected by the lack of CRY, ITP and PDF, although their amplitudes were reduced. In contrast, the amplitude of the rhythm of *clk* was higher in *cry*^01^ and *cry > itpRNAi* than in Canton S flies. The lack of CRY and ITP increases *per* and *tim* expression at the end of the night and at the beginning of the day. It means that accumulation of CRY during the night in the photoreceptors, represses *per* and *tim* transcription at the end of the night and the beginning of the day, probably by inhibiting the *clk* expression at ZT16. These results show that CRY might be a repressor of the molecular clock in the retina, as it has already been reported for peripheral oscillators (Collins et al., 2006). It is also possible that in the *cry*^01^ mutant the molecular oscillators are not entrained and because of their desynchronisation the amplitude of oscillations is low. The expression of *cry* in the retina was only slightly changed by the lack of PDF and ITP by increasing the mRNA of *cry* during the night in the *Pdf* ^0^ mutant and after the silencing of *itp*. The obtained results confirm the finding of other authors suggesting that the retina possesses an autonomous clock (Cheng and Hardin, 1998).

In the lamina, the clock genes also have a cyclic expression but the daily patterns of their expression are different than in the retina. Moreover, the rhythms of *per* and *tim* expression are regulated by both PDF and ITP and in case of *per*, but not *tim*, they changed in the phase in the *cry*^01^ mutant.

The similar daily expression in the lamina and retina was found only in the case of *clk* and *cry*. The rhythm of *cry* expression in the lamina is affected by PDF but its mRNA level was very low in glial cells and not detected in the L2 cells. It suggests that the clock in the glial cell may not require CRY, but that expression of *cry* in the lamina may originate from the photoreceptor terminals in the lamina. In turn, the *per* and *tim* mRNA rhythms in the lamina may reflect the expression of *per* and *tim* in the epithelial glial cells that is modified by specific *per* and *tim* expression in neurons, for example in the L2 monopolar cells.

In contrast to clock genes, the daily rhythms of *Atpα* and *nrv2* expression in the retina are controlled by the pacemaker, since in the *Pdf* ^0^ mutant and after silencing *itp*, the pattern of both rhythms was changed or the rhythm was disrupted as in the case of *Atpα* in *cry > itpRNAi* flies. The rhythm of the sodium pump α subunit confirms that the activity of this ion pump is cyclically regulated, not only in the lamina, but also in the retina by changes to the *Atpα* expression. In turn, the rhythmic expression of *nrv2* only in the retina, suggests that this protein might be involved in other cyclic functions of Na^+^/K^+^-ATPase rather than in ion pumping. It has been reported that Na^+^/K^+^-ATPase participates in the formation of cell junctions and the β subunit forms bridges between cells (Paul et al., 2007; Vagin et al., 2012). In contrast, in the lamina, glial and L2 cells, only the expression of *Atp*α was rhythmic and the pattern of this rhythm was similar to the daily rhythm of the ATPα protein detected in the lamina glia (Damulewicz et al., 2013). However, the expression of *Atp*α was different in the L2 cells. The mRNA of *Atp*α and ATPα protein peak at ZT13 in neurons, while in the glia they peak twice at ZT1 and ZT13 (Damulewicz et al., 2013). In the lamina the expression of all genes studied was changed in *cry*^01^, *Pdf* ^0^ mutants and in *cry > itpRNAi* line, indicating that rhythms in the lamina are controlled by several oscillators, located in the retina, the pacemaker and in the lamina itself. Moreover, PDF and ITP neuropeptides are transmitters of circadian information from the pacemaker to the lamina. It confirms our earlier studies which showed that the circadian rhythms of two proteins in the lamina, the α subunit of Na^+^/K^+^-ATPase and BRP, are also regulated by these three types of oscillators (Damulewicz et al., 2013; Górska-Andrzejak et al., 2013). In addition, the oscillation of ATPα level is affected by the lack of both PDF and ITP.

Another ccg that was studied in the retina and lamina was *brp*. The presynaptic protein BRP, encoded by this gene, shows the circadian rhythm in abundance in the lamina, with two peaks, in the morning and in the evening (Górska-Andrzejak et al., 2013). Here, we found that *brp* expression oscillates in both the retina and lamina, however, the pattern is different than that found in the case of BRP protein. This can be explained by the fact that the protein was measured only in tetrad synapses in the lamina, while mRNA was examined in all lamina neurons. In the retina, *brp* mRNA was highest at ZT16 and this rhythm was unchanged in the *cry*^01^ mutant and by the lack of PDF and ITP. In the lamina, however, the daily pattern of *brp* expression was very different when CRY, PDF or ITP were eliminated or as in the case of ITP decreased.

In two selected cell types, namely: L2 interneurons and glial cells, we detected the robust rhythm of *Pdfr* mRNA with a peak at ZT1, suggesting that this gene is another ccg and that the rhythms in the lamina, at least in the L2, and in glial cells, are controlled by PDF and the cyclic expression of its receptors.

In the present study, we showed that clock genes are expressed differently in the retina and in the first optic neuropil (the lamina), of the brain optic lobe.

Moreover, we showed that cells which do not contain circadian clocks cyclically express genes and show circadian rhythms in cellular processes. In both cell types, L2 interneurons and glial cells, the α subunit of the sodium pump shows the cyclic expression. It suggests that many cellular processes in neurons and glial cells might be controlled over time by the cyclic activity of the sodium pump. In addition to cellular processes in neurons and glia, synaptic transmission is also controlled by the clock, since expression of *brp* and level of BRP (Górska-Andrzejak et al., 2013) as well as other genes and their proteins in synapses, oscillate during a 24 h period (Krzeptowski et al., 2014).

On the basis of our results and the findings of other authors (Gorostiza et al., 2014), we suggest that the pacemaker controls the circadian rhythms in the brain by cyclic remodeling of synaptic contacts, between the clock and target neurons. However, in distant brain tissues, as in the lamina, circadian rhythms are regulated by neuropeptides released from the pacemaker neurons and by the daily expression of PDF receptors in neurons and glial cells. Moreover, peripheral oscillators, as glial cells, locally shape the circadian rhythms of the structure of neurons and synapses in particular brain regions and cell types.

## Conflict of Interest Statement

The authors declare that the research was conducted in the absence of any commercial or financial relationships that could be construed as a potential conflict of interest.
